# Did the mental health and well-being of young people increase after the COVID-19 vaccination campaign period? A cross-sectional multicentre study in Austria and Turkey

**DOI:** 10.1007/s12144-023-04366-x

**Published:** 2023-02-21

**Authors:** Zeliha Özlü-Erkilic, Oswald D. Kothgassner, Thomas Wenzel, Andreas Goreis, Anthony Chen, Veysi Ceri, Aylin Fakhr Mousawi, Türkan Akkaya-Kalayci

**Affiliations:** 1 Department of Child and Adolescent Psychiatry, Outpatient Clinic of Transcultural Psychiatry and Migration Induced Disorders in Childhood and Adolescence, Medical University of Vienna, Währinger Gürtel 18-20, 1090 Vienna, Austria; 2 Postgraduate University Program Transcultural Medicine and Diversity Care, Medical University of Vienna, Spitalgasse 23, 1090 Vienna, Austria; 3 Department of Child and Adolescent Psychiatry, Medical University of Vienna, Währinger Gürtel 18-20, 1090 Vienna, Austria; 4 Department of Psychiatry and Psychotherapy, Medical University of Vienna, Währinger Gürtel 18–20, 1090 Vienna, Austria; 5 Scientific Section on Psychological Aspects of Torture and Persecution, World Psychiatric Association (WPA), 1226 Thônex, Switzerland; 6 Faculty of Health Sciences, Department of Child Development, Batman University, Üniversitesi, Merkez Kampüsü, 72060 Batman, Turkey

**Keywords:** COVID-19, Mental health, Psychological well-being, Vaccination, Young people, Austria, Turkey

## Abstract

In 2019, the global Coronavirus (COVID-19) pandemic and associated prevention measures affected the mental health and psychological well-being of young people with and without a migratory background. The present study aimed to compare the mental health and psychological well-being of migrant and non-migrant young people before and after the COVID-19 vaccination campaign period in two countries which had different policies to deal with the pandemic. The “Psychological General Well-being” of young people and their experiences during the pandemic were investigated using an anonymous online survey during two pandemic waves before the vaccination campaign and 6 months after its start. A majority of the 6154 participants (aged between 15 and 25 years in all study groups) reported a decrease of mental health from the time period before the vaccination (BV) to after the vaccination (AV) campaign (*b* = 0.27, *p* < .001). This association was higher in females (*b* = 0.04, *p* = 0.008) and in youth with financial problems (*b* = 0.13, *p* < .001). Furthermore, this decrease was more pronounced in people ≤17 years old (40% to 62%) than in those >17 years (59% to 67%). Contrary to expectation, vulnerable groups such as the economically disadvantaged, younger, and female participants did not experience a significant easing of the pandemic’s psychological burden AV. Vaccination campaigns should continue emphasizing the beneficial effects of COVID-19 vaccination on general well-being, but with an acknowledgement that the road to recovery is still a long one. Concurrently, free access to psychological treatment and financial support should be offered, especially for vulnerable groups.

**Supplementary Information:**

The online version contains supplementary material available at 10.1007/s12144-023-04366-x.

## Introduction

At the beginning of 2020, the Coronavirus disease (COVID-19) which began in 2019 had already quickly spread globally (Bono et al., 2021), and in a very short time many people were affected by illness or death caused by COVID-19 (World Health Organization, 2020). The adverse outcomes of the COVID-19 pandemic such as loss of work, social isolation, care-giving burdens, food insecurity, and racialized discrimination led to increased mental health problems throughout the general population (Fang et al., 2021; Hertz-Palmor et al., 2021; Pancani et al., 2021). Young people seemed especially affected by implemented lockdown measures, evidenced by the increase of mental health problems among young people, particularly females, during the COVID-19 pandemic (Dale et al., 2021; Pieh et al., 2021). Similarly, the study of Rossi et al., 2020 reported that, in Italy, being female and young were risk factors for a more pronounced deterioration of mental health status during the COVID-19 pandemic (Rossi et al., 2020). Furthermore, social distancing and homeschooling also negatively impacted the mental health of children and adolescents (Nocentini et al., 2021; Ravens-Sieberer et al., 2021; Viner et al., 2022). Levels of psychological distress, suicidal ideation, and general anxiety increased noticeably after the outbreak of the COVID-19 pandemic (Daly & Robinson, 2021; O'Connor et al., 2020), but mental health services were restricted to emergency treatment in many countries, during the most stringent lockdown periods.

Health problems disproportionately affect vulnerable groups such as migrants and minorities (Raisi-Estabragh et al., 2020). In European countries as well as outside of Europe, migrants are usually at a much higher risk of COVID-19 infection compared to native populations (Guttmann et al., 2020; OECD, 2020). This might be due to factors like poor housing conditions, a higher risk of poverty, and dependence on jobs where working from home is not possible, resulting in exposure to the public (OECD, 2020).

In the USA and the UK, the mortality risk among migrant groups was 10-15% higher than for the native population (Public Health England (PHE), 2020; US Centers for Disease Control and Prevention, 2020). Furthermore, both adult and minor migrants already have been shown to suffer more from mental health problems when compared to native populations, even before the pandemic. It is regrettable that this gap has only widened during the COVID-19 pandemic, especially for young migrants (Ravens-Sieberer et al., 2021). While the migrant population in recent studies was younger than their native counterparts, in general, migrants had higher infection risk and mortality (OECD, 2020). In summary, during the COVID-19 pandemic, migrants suffered disproportionately when compared to native populations (OECD, 2020).

In Austria and Turkey, different lockdown restrictions were set to overcome the COVID-19 pandemic, which in conjunction with the stress of pandemic-related factors led to a negative impact on the mental health of both young native adults and young migrants (Akkaya-Kalayci et al., 2020; Galea et al., 2020; Özlü-Erkilic et al., 2021). The authors observed increases in stress, anxiety, and depression (Gualano et al., 2020; Salari et al., 2020). With this setting, it was hoped that increased availability of free COVID-19 vaccinations, intended to begin the journey of returning to normal life by slowing the spread of disease and reducing the need for restrictions, would have resulted in some improvement in mental well-being (Bilge et al., 2021). It is expected that COVID-19 vaccines will also significantly reduce health risks in general, and in the long term improve economic and social outcomes associated with the pandemic, with a resulting benefit for mental health (Perez-Arce et al., 2021). Some studies have already demonstrated that widespread availability of the COVID-19 vaccine has led to a substantial improvement in mental health (Bilge et al., 2021; Koltai et al., 2021; Perez-Arce et al., 2021). Like other countries around the world, Austria and Turkey have tried diverse strategies to vaccinate their populations and fight against the anti-vaccine movements, with their campaigns starting at similar times.

In December of 2020, the first COVID-19 vaccine was approved in the European Union (EU), after accelerated testing and approval procedures (European Medicines Agency, 2020). Now, in Austria and the EU there are currently two mRNA vaccines and two vector vaccines which are approved (European Medicines Agency, 2021). The first COVID-19 vaccinations were performed in Austria and Turkey in December, 2020. Initially, the vaccines were only provided to adults, specifically elderly adults and people in at-risk groups. During this first phase of COVID-19 vaccination, it was not permitted to children and youths under the age of 18 years. In both countries (Austria and Turkey) there were strict timepoints for vaccination booster shots, which depended on the type of vaccine and the age of the vaccinated person. As the vaccination booster period varied between 3 and 12 weeks, there was no general timeline for vaccination strategy in both countries. In Austria as well as in Turkey, diverse campaigns via television or social media were undertaken to motivate the population to receive COVID-19 vaccinations. Informatic videos were deployed to prevent misinformation about the COVID-19 vaccination and its outcomes.

### Aim of the study

The purpose of the present study was to test the hypothesis that the mental health and well-being of young people and vulnerable groups would improve after the main vaccination campaign period in Austria and in Turkey during the ongoing COVID-19 pandemic.

Because vaccination as an answer to the COVID-19 pandemic improved the mental health of adults (Bilge et al., 2021; Perez-Arce et al., 2021), the present study examined whether these vaccination campaigns had the same effect among young people.

To analyze the mental health and well-being of young people and vulnerable groups, we compared youths with and without a migration background living in Austria and Turkey, resulting in a total of four groups in the present study.

This allowed us to analyze the effect of vaccination among different populations living in the same area (native versus migrant youths) in Austria and Turkey. We hypothesized that there would be differences in mental health and well-being among youths with and without migration background after the vaccination campaign period, as general differences were already present between natives and migrants during the COVID-19 pandemic before the vaccination period. Migrant populations suffered disproportionately when compared to native populations, as that migrants generally had higher infection and mortality rates (OECD, 2020).

Migration status has, as noted before, been identified as a significant factor in predicting well-being during the pandemic and lockdown (Fang et al., 2021; Kluge et al., 2020), and the percentage of migrants is high in both countries, which have been affected differently and adopted different lockdown strategies. We therefore also aimed to explore two important factors – country and migration status and their respective influence on the psychosocial impact of the pandemic before and after recent vaccination campaigns.

**Fig. 1:**
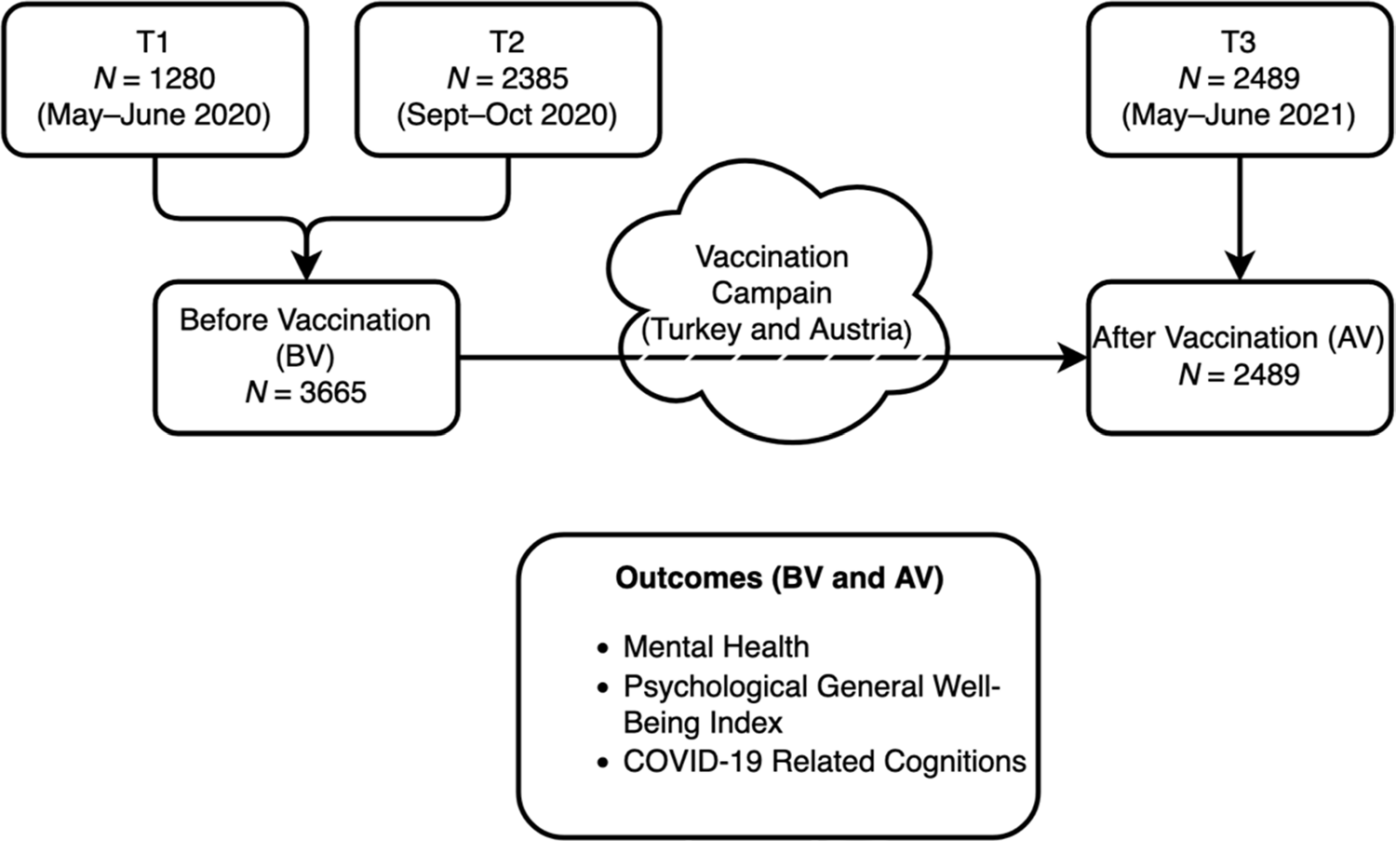
Graphical depiction of the study design

In summary, the focus of the present study was to compare the times before and after the COVID-19 vaccination was available, as well as to examine its impact among young people with different backgrounds (migrant or native) in two countries (Austria and Turkey). See Fig. 1 for a graphical depiction of the study design.

## Methods

### Participants

Young people aged between 15 and 25 years, who lived in Austria and Turkey, were included in the study via an anonymous online survey, which was available in German or Turkish. Inclusion criteria were that the participant was 1) living inside Austria or Turkey, 2) had sufficient German or Turkish language skills to understand the survey content, and 3) be of an age between 15 and 25 years. The survey was then presented in their preferred language. The study participants were split into four groups based on their self-reported backgrounds as Austrian natives, Austrian migrants (people with migration background in the first or second generation, living in Austria), Turkish natives, or Turkish migrants (people with migration background, in the first or second generation, living in Turkey), independent of their citizenship status. The last factor was not considered, as a large part of the migrant population living in Austria and Turkey possess dual citizenships. The participants were assessed independently and cross-sectionally at three timepoints, resulting in similar groups of different individuals, as due to data safety restrictions, it was not possible to follow-up specific individuals from T1/T2 to T3. Because the online survey was anonymous, tracking an individual’s responses across the three survey was not possible.

As mentioned before, the study participants were split into four groups, therefore the results of the present study are based on aggregate data and not a specific person-to-self comparison. The study subjects were recruited in the same way, via the same social media channels and e-mail contacts to institutions working with young people (e.g., youth centers, schools) for all surveys.

### Procedures

The anonymous online survey (SoSci Survey) was administered to adolescents and young adults at three timepoints, with two corresponding to two infection “waves”: first at the height of the first pandemic (T1) from May 22^nd^ to June 19^th^, 2020, and second at the beginning of the second wave (T2) from September 11th to October 23^rd^, 2020, before the beginning of the vaccination campaign. The third assessment was conducted between May 18^th^ and June 8^th^, 2021, in the above two countries, after vaccines had been available in both countries for about half a year. We analyzed the psychological well-being of migrant and non-migrant young people aged between 15 and 25 years after the COVID-19 vaccination campaign period.

Austria and Turkey followed different pandemic control strategies and have different cultures, but both suffered from high COVID-19 prevalence. For the present study we compared the mental health and psychological well-being of young people living in Austria and Turkey with and without migration background before and after the main vaccination campaigns. In the initial phase, the COVID-19 vaccination was only available for elderly adults and those belonging to certain at-risk groups such as people who have diabetes, heart disease, etc., or for staff working in the health-care system. This facet of the vaccination campaign was the same in both Austria and Turkey. As vaccines had been seen as a part of the solution for the still ongoing COVID-19 pandemic, it was quite important to also analyze the psychosocial impact of the vaccination campaign for young people. Therefore, we analyzed the differences in mental health and psychological well-being before and after the initial vaccination campaign among participants 15-25 years of age, split into those under 18 and those over 17.

Between May 18^th^ and June 8^th^, 2021, we conducted this online survey via social media and e-mails, after the COVID-19 vaccination had been available in Austria and Turkey for approximately half a year.

For the comparison, we merged the two first data sets on psychosocial well-being during waves 1 and 2 (T1 and T2), as they were both before the vaccination campaign period (BV). The BV period therefore includes participants who filled out our survey from May to June 2020 (originally: T1) and from September to October 2020 (T2). We compared results to a similar sample of young adults taken after the vaccination campaign period (AV). We assessed mental health, psychological well-being, experiences, and concrete individual fears and cognitions related to the pandemic in migrants and native populations in both countries. See (Akkaya-Kalayci et al., 2020) and (Özlü-Erkilic et al., 2021) for a detailed description of the T1 and T2 timepoints.

### Instruments

At all three timepoints, we used the validated German (DuPuy et al., 1993) and Turkish (Ay et al., 2010) versions of the Psychological General Well-Being Index (PGWBI) (Dupuy, 1984) which consists of 22 items on 6-point Likert-type scales, divided into six subscales: Anxiety, Depressed mood, Positive well-being, Self-control, General health, and Vitality. The PGWBI captures the general well-being during the last month, but for the present study, we extended the query to refer to the previous two months to cover the quarantine time at the onset of the pandemic and the 2^nd^ wave, as well as time AV. Importantly, and in line with the manual, all subscales of the PGWBI were scored such that higher values indicated greater well-being (i.e., a higher score in the anxiety and depression subscales means presence of a smaller number of symptoms of anxiety and depression). The reliability was good or acceptable for the subscales of the PGWBI in the current study (Macdonald’s ω: anxiety = .91, depressed mood: .88, personal well-being = .82, self-control = .81, general health = .72, and vitality = .83).

The psychological impact of the COVID-19 pandemic was further measured using socio-demographic data and questions covering self-reported individual experiences and changes during the COVID-19 pandemic and quarantine periods. The items in this additional questionnaire developed for the present research included one item on the subjective personal estimate of the deterioration of mental health because of the COVID-19 pandemic: “Has your mental health changed since the pandemic?” (response options: 1 = improved, 2 = deteriorated, 3 = unchanged), and a proxy item for socioeconomic status: “Do you experience financial problems because of the COVID-19 pandemic?” (response options: 1 = yes, 0 = no).

Furthermore, five single items on COVID-19 related conditions were assessed: 1) rumination; 2) fear of becoming infected; 3) fear relating to infection of a family member; 4) belief that pandemic restriction measures were exaggerated and 5) the individuals’ personal expectation of COVID-19 risks all on 5-point Likert-type scales, ranging from 1 = never to 5 = always. The same questionnaires were used at T1, T2, and T3. All study participants provided electronic informed consent together with parental consent before starting the online survey. The online survey took approximately 10 minutes to complete. Only data with a complete set of responses were included.

The study was conducted in accordance with the Declaration of Helsinki, and the protocol was approved by the Ethics Commission of the Medical University of Vienna (protocol number: EK 1488/2020).

### Statistical analysis

Data were analyzed using R version 4.0.3 (R Core Team, 2020). Multilevel models (MLMs) were created to simultaneously compare the four study groups at two sampling times while taking the variability of each participants’ migrant group into account (i.e., Austrian native, Austrian migrant, Turkish native, or Turkish migrant) (i.e., random intercept models). MLMs were fitted using the lm4 package (Bates et al., 2015) with *p*-values supplied by the lmerTest package (Kuznetsova et al., 2017). For each of the outcomes (i.e., deterioration of mental health, subscales of the PGWBI, and subscales of the COVID-19 questionnaire), we fitted a random effects model, which also incorporated the potential predictors of gender and reported financial problems. Furthermore, the variable of age (below 18 or above 17 years) was computed to serve as a proxy for those eligible to receive a vaccination. Interactions of migrant group with before/after vaccination time period and age category with before/after vaccination time period were analyzed and followed up by simple effects analyses to compute potential differences between the four groups over time. This analysis strategy allowed us to a) compare our outcomes across timepoints, countries, and migration status via interactions and b) identify potential interactions with age or financial difficulties in any group/migration status combination (i.e., simple effects).

## Results

### Participants and sample composition

In total, we analyzed the data of *N* = 6154 participants that fit inclusion criteria and received informed consent as outlined above. In the time before the main vaccination campaign (aggregated T1 and T2 = BV), *n* = 3665 participants filled out the survey, while *n* = 2489 participated at T3 AV (after the main vaccination campaign). At BV, *n* = 1056 were Austrian natives, *n* = 380 were Austrian migrants, *n* = 1704 were Turkish natives, and *n* = 525 were Turkish migrants. At T3, 846 participants were Austrian natives, 491 were Austrian migrants, 1000 were Turkish natives, and 152 were Turkish migrants. Of the Austrian migrants, *n* = 408 (*n* = 158 at BV; *n* = 250 at AV) were born outside of Austria (i.e., first-generation immigrants), and *n* = 455 (*n* = 222 at time BV; *n* = 233 at time AV) were born in Austria with at least one parent born in another country (i.e., second-generation immigrants). Of those born outside of but now residing in Austria, 19% were born in former Yugoslavian counties, 16% in Turkey, 10% in Afghanistan, 8% in Syria, 5% in Chechnya, and the remaining 35% in a wide array of over 40 countries. Of the migrants residing in Turkey, 17 (*n* = 11 at time BV; *n* = 6 at time AV) were first-generation immigrants, whereas the remainder (*n* = 660; *n* = 514 at time BV: *n* = 146 at time AV) were second-generation immigrants. Those born outside of Turkey were primarily from Germany (*n* = 6), Iran (*n* = 2), and nine other countries were represented with one participant each (see Table 1 for detailed sample characteristics, including age and gender distribution, across all four groups before and after the main vaccination campaign period (see Supplementary Tables 1 and 2 for descriptive baseline).

**Table 1:** Description of reported mental health in the different groups at the two timepoints (BV, AV) before Vaccination Campaign Period = May 22–June 19 and September 11–October 23, 2020 (T1, aggregated with T2, BV), and after Vaccination Campaign Period: May 18–June 8, 2021

	Before Vaccination Campaign Period (*N* = 3665)			After Vaccination Campaign Period (*N* = 2489)		
	Age *M* (*SD*)	Decrease in reported Mental Health (% yes)	Financial problems (% yes)	Age *M* (*SD*)	Decrease in reported Mental Health (% yes)	Financial problems (% yes)
Sample Austria (*n* = 2773)	20.16 (3.44)	32.52	13.58	19.40 (3.27)	56.85	17.03
Austria Native (*n* = 1902)	20.36 (3.49)	33.43	11.93	20.50 (3.57)	58.31	11.42
Male (*n* = 592)	20.04 (3.59)	22.29	9.24	19.25 (3.02)	48.48	10.61
Female (*n* = 1276)	20.51 (3.44)	38.03	12.59	19.60 (3.58)	62.44	11.34
Austria Migrant (*n* = 871)	19.60 (3.22)	30.00	18.16	19.24 (3.01)	54.57	25.78
Male (*n* = 341)	19.59 (3.15)	25.17	16.08	19.11 (2.64)	47.56	27.61
Female (*n* = 519)	19.61 (3.28)	32.34	19.15	19.33 (3.28)	58.94	24.80
Sample Turkey (*n* = 3381)	21.81 (2.98)	69.94	49.89	22.11 (2.99)	75.90	51.81
Turkey Native (*n* = 2704)	21.71 (3.01)	68.78	49.41	22.02 (3.01)	76.01	49.26
Male (*n* = 1953)	21.77 (2.90)	70.32	49.56	22.16 (2.90)	76.44	49.85
Female (*n* = 740)	21.60 (3.19)	66.02	49.20	21.32 (3.44)	73.88	45.52
Turkey Migrant (*n* = 677)	22.12 (2.85)	73.71	51.43	22.71 (2.80)	75.19	67.97
Male (*n* = 445)	21.95 (2.73)	77.34	53.17	22.36 (2.84)	80.61	65.31
Female (*n* = 232)	22.42 (3.02)	67.53	48.45	23.76 (2.41)	58.06	76.67

*n* = 37 participants reported “diverse” as gender, *n* = 29 did not give information on their gender

### Effects of the COVID-19 pandemic on mental health and psychological well-being before and after the main vaccination campaign period

Analyses of the dynamic changes in subjective assessment of mental health by the participant (our one-item indicator: “has your mental health deteriorated because of the COVID-19 pandemic?”) revealed that overall, more people reported a decrease of mental health from BV to AV (*b* = 0.27, *p* < .001). This association was stronger when financial problems were reported at any time (*b* = 0.13, *p* < .001), and when the gender was female (*b* = 0.04, *p* = 0.008). Regarding changes from time BV and time AV, native Austrians reported the highest decrease in mental health (33% to 58%, *p* < .001), followed by Austrian migrants (30% to 55%, *p* < .001), Turkish natives (69% to 76%, *p* = <.001). Turkish migrants did not indicate a significant decrease in mental health from wave time BV and time AV (73% to 75%, *p* = .429). A significant negative interaction of age group with time AV (*b* = − 0.08, *p* = .014) indicated that the decrease in mental health was associated with age. Both age groups reported a decrease from BV to AV, but the decrease was higher in people <18 years old (40% to 62%) than in those >17 years (59% to 67%) as those >17 years old already reported a higher rate of mental health deterioration BV. This association between age group and time did not differ across the four groups as the three-way interaction of group x time x age group was not significant (*F* = 1.57, *p* = .195).

**Fig. 2:**
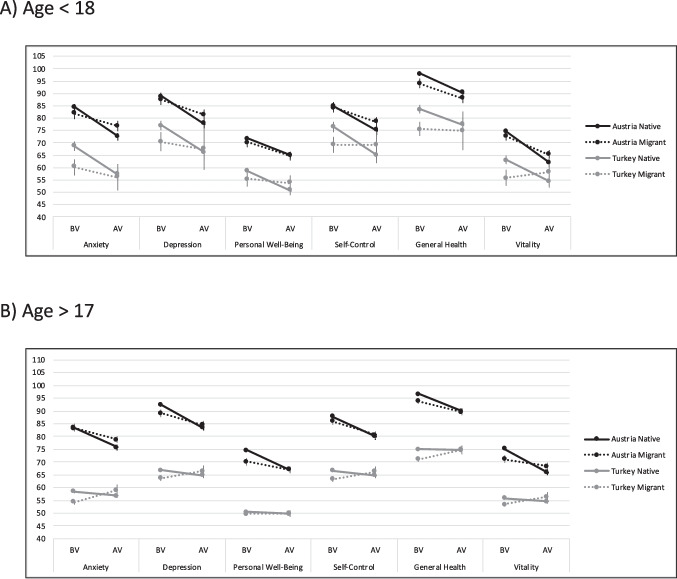
Subscales of the PGWBI for the four groups for participants that were (**A**) below 18 years old, and (**B**) above 17 years old, separated by waves before vaccination campaign (BV) and after vaccination campaign (AV; *M* and *SEM*)

The analyses for the subscales of the PGWBI are reported in detail in Table 2 and Fig. 2, split by age group (Fig. 2A and B). Overall, without taking individual countries or migrant status into account, the researchers’ analyses showed that the scores of all the subscales denoting psychological well-being decreased (i.e., indicating more symptoms/impairment in those subscales) from time BV to AV, as all coefficients are negative and significant (see Table 2 for all coefficients). The subscales of the PGWBI furthermore indicated more symptoms and impairment by people who reported financial problems (all coefficients of financial problems were negative, *p*s < .001).

**Table 2:** Results of multilevel models of the subscales of the Psychological General Well-Being Index

	Anxiety		Depression		Personal Well-Being		Self-Control		General Health		Vitality	
Predictors	*b*	*p*	*b*	*p*	*b*	*p*	*b*	*p*	*b*	*p*	*b*	*p*
(Intercept)	85.84	<0.001	90.05	<0.001	73.12	<0.001	86.47	<0.001	98.79	<0.001	75.72	<0.001
Time AV	−11.45	<0.001	−10.73	<0.001	−6.81	<0.001	−9.93	<0.001	−7.18	<0.001	−12.98	<0.001
Austria Migrant	−2.51	0.575	−1.29	0.604	−1.59	0.678	−0.85	0.978	−3.77	0.868	−1.87	0.918
Turkey Native	−13.83	0.002	−9.33	<0.001	−12.21	0.001	−6.55	0.827	−12.36	0.585	−9.78	0.590
Turkey Migrant	−21.49	<0.001	−15.06	<0.001	−14.84	0.001	−13.12	0.664	−19.90	0.383	−16.39	0.372
Age > 17	−0.58	0.694	4.22	0.009	3.26	0.005	3.27	0.049	−0.83	0.537	0.97	0.481
Female	−0.75	0.231	0.15	0.830	−0.94	0.056	−0.34	0.627	−0.23	0.691	−0.23	0.698
Financial Problems	−9.63	<0.001	−11.24	<0.001	−6.18	<0.001	−9.73	<0.001	−7.91	<0.001	−8.15	<0.001
Time AV x Turkey Native	6.75	0.040	5.18	0.145	1.52	0.556	4.45	0.226	1.41	0.636	5.48	0.072
Time AV x Turkey Migrant	−0.53	0.881	−0.99	0.796	−1.31	0.637	−2.29	0.565	0.48	0.883	3.63	0.270
Time AV x Age > 17	10.33	0.230	8.35	0.369	6.91	0.305	13.04	0.176	15.06	0.054	17.89	0.025
Austria Migrant x Age > 17	3.75	0.111	1.54	0.543	−1.13	0.540	1.88	0.476	0.52	0.808	3.49	0.109
Turkey Native x Age > 17	3.27	0.241	−1.33	0.660	−2.58	0.237	−0.48	0.878	1.46	0.564	−1.63	0.527
Turkey Migrant x Age > 17	−7.68	0.001	−12.34	<0.001	−10.23	<0.001	−11.31	<0.001	−6.31	0.002	−6.68	0.002
Austria Migrant x Time AV x Age > 17	−4.13	0.288	−9.65	0.021	−8.15	0.007	−8.06	0.064	−2.69	0.446	−2.52	0.484
Turkey Native x Time AV x Age > 17	−2.48	0.536	0.57	0.895	3.90	0.214	−0.66	0.882	1.81	0.619	1.95	0.599
Turkey Migrant x Time AV x Age > 17	6.31	0.106	7.79	0.065	8.31	0.007	8.32	0.057	5.83	0.100	4.32	0.232
Time AV x Turkey Native	3.80	0.672	6.29	0.517	2.12	0.762	−0.45	0.965	−3.48	0.669	−4.00	0.630

All factorial variables were dummy coded with the reference categories: AV (after vaccination campaign) vs. BV (before vaccination campaign); Austria migrant, Turkey native, and Turkey migrant vs. Austria native; Age < 18 vs. age > 17; female vs. male; financial problems

Regarding differences between the two countries, we found a stable pattern of results for all subscales of the PGWBI: In every subscale, Austrian natives reported the least impairment, followed by Austrian migrants, and then followed by Turkish natives (all *p*s of simple effects analyses < .001). Turkish migrants, in contrast, reported an improvement in the subscales of anxiety (*p* = .006), depression (*p* = .003), self-control (*p* = .028), general health (*p* = .009), and vitality (*p* = .025). Only on the subscales of personal well-being and self-control did Turkish migrant scores not change from time BV to AV (*p*s = .548 and .065, respectively). Regarding effects of the age group (and therefore, vaccination), there were significant interactions of age group with timepoint. These indicated that all scores decreased more (i.e., more symptoms) from time BV to time AV in those <18 than in those >17 on the subscales of depression, personal well-being, self-control, and vitality. Older people reported an increase in symptoms over time that was not as high compared to younger people, however, there was still some impairment. Only on the subscales of anxiety and general health was there an increase in symptoms for young people (anxiety: *b* = 4.31, *p* = .001; general health: *b* = 4.31, *p* = .001) but not for the <18 age group (anxiety: *b* = 1.27, *p* = .120; general health: *b* = 1.11, *p* = .132), such that symptoms did not change in older people after the vaccination campaign (AV). Again, these associations between age group and time did not differ across the four migrant groups as the three-way interaction of group with time and age group was not significant in any of the subscales (see Table 2).

Additional exploratory analyses of all mental health outcomes with migrant status, choosing first versus second-generation migrant status as predictors in the two migrant groups, revealed that in Austrian migrants, those who were born outside of Austria reported a significantly higher deterioration in mental health status (17%) than second-generation immigrants in Austria (14%; *b* = 0.11, *p* = .042). No other significant associations of migration status with any of the PWB subscales were found (all *p*s > .338).

### Differences regarding COVID-19 related cognitions

**Fig. 3:**
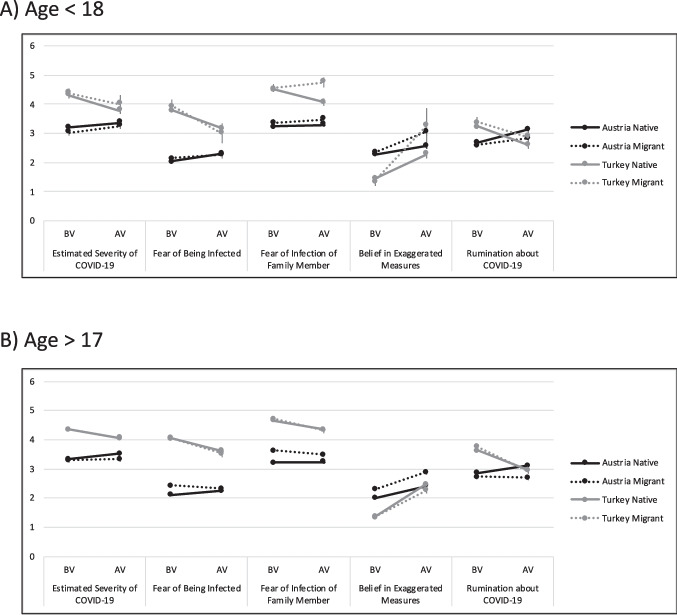
Subscales of the COVID-19 related cognitions for the four groups for participants that were (**A**) below 18 years old, and (**B**) above 17 years old, separated by waves before vaccination campaign (BV) and after vaccination campaign (AV; *M* and *SEM*)

The analyses for the questionnaire’s subscales pertaining to cognitions and opinions related to the pandemic are depicted in Table 3 and Fig. 3. Again, as demonstrated in Fig. 3, an increase in a number of items could be seen across almost all four groups of young people from BV to AV. The main effects of time (i.e., AV compared to BV) clearly showed that as the pandemic progressed, the fear of the individual being infected (*b* = 0.27, *p* = .008) increased across all four groups without taking individual country or migrant status into account. However, the estimated severity of COVID-19 infection (*b* = 0.16, *p* = .070) and the fear of the infection of a family member did not change over time (*b* = 0.05, *p* = .590). Reports of beliefs of measures against COVID-19 as being exaggerated were also higher at AV (*b* = 0.27, *p* < .007). Ruminations about COVID-19 also increased across all four groups (*b* = 0.42, *p* < .001). Ratings of all five items were higher when participants reported financial problems (all *p*s < .013) and—except for estimated severity and fear of being infected, where the effect was not found—when the sex was female (all *p*s < .004, see Table 3).

**Table 3:** Results of multilevel models of the subscales of the COVID-19-related cognitions questionnaire

	Estimated Severity of COVID-19		Fear of Being Infected		Fear of Infection of a Family Member		Belief in Exaggerated Measures		Rumination about COVID-19	
Predictors	Estimates	*p*	Estimates	*p*	Estimates	*p*	Estimates	*p*	Estimates	*p*
(Intercept)	3.20	<0.001	2.05	0.165	3.13	<0.001	2.19	<0.001	2.63	<0.001
Wave 2	0.16	0.070	0.27	0.008	0.05	0.590	0.27	0.007	0.42	<0.001
Austria Migrant	−0.17	0.382	0.10	0.963	0.13	0.483	0.07	0.731	−0.11	0.479
Turkey Native	1.11	<0.001	1.72	0.410	1.28	<0.001	−0.86	<0.001	0.51	<0.001
Turkey Migrant	1.16	<0.001	1.84	0.378	1.29	<0.001	−0.93	<0.001	0.66	0.001
Age > 17	0.15	0.026	0.07	0.386	−0.03	0.650	−0.30	<0.001	0.17	0.020
Female	−0.02	0.399	−0.04	0.205	0.14	<0.001	0.13	<0.001	0.09	0.004
Financial Problems	0.07	0.013	0.18	<0.001	0.16	<0.001	0.12	<0.001	0.17	<0.001
Time AV x Austria Migrant	0.05	0.763	−0.18	0.287	0.06	0.719	0.40	0.017	−0.22	0.160
Time AV x Turkey Native	−0.71	<0.001	−0.92	<0.001	−0.49	0.004	0.60	0.001	−1.04	<0.001
Time AV x Turkey Migrant	−0.58	0.139	−1.30	0.004	0.16	0.698	1.63	<0.001	−0.96	0.021
Time AV x Age > 17	0.01	0.958	−0.13	0.288	−0.00	0.996	0.15	0.209	−0.19	0.097
Austria Migrant x Age > 17	0.12	0.366	0.22	0.131	0.28	0.037	0.23	0.111	−0.03	0.822
Turkey Native x Age > 17	−0.14	0.172	0.13	0.272	0.17	0.126	0.21	0.069	0.22	0.047
Turkey Migrant x Age > 17	−0.20	0.268	0.02	0.916	0.19	0.298	0.30	0.134	0.20	0.286
Austria Migrant x Time AV x Age > 17	−0.19	0.287	−0.11	0.608	−0.27	0.169	−0.22	0.285	−0.08	0.695
Turkey Native x Time AV x Age > 17	0.24	0.180	0.34	0.102	0.14	0.446	0.12	0.550	0.14	0.447
Turkey Migrant x Time AV x Age > 17	0.14	0.742	0.61	0.200	−0.61	0.158	−1.18	0.010	−0.10	0.810

All factorial variables were dummy coded with the reference categories: AV (after vaccination campaign) vs. BV (before vaccination campaign); Austria migrant, Turkey native, and Turkey migrant vs. Austria native; Age < 18 vs. age > 17; female vs. male; financial problems indicated vs. not indicated

Regarding differences between the two countries, results for the estimated severity of COVID-19 and fear of being infected had similar ratings: Austrian natives had significantly higher estimates at time AV than at time BV (*p*s < .002), Austrian migrants did not report changes (*p*s = .293 and .440), and Turkish natives and Turkish migrants reported a decrease in these two items (*p*s < .003). The fear of infection of a family member did not change over time in Austrian natives and migrants (*p*s > .414) and was lower at wave 2 in Turkish migrants, followed by Turkish natives (*p*s < .002). Agreement to the statement that measures against the pandemic were exaggerated increased over time in all groups and was highest in the group of Turkish natives, followed by Turkish migrants, Austrian migrants, and Austrian natives (all *p*s < .001). Ruminations about COVID-19 increased only in Austrian natives (*p* < .001) yet showed no difference over time in Austrian migrants (*p* = .398). A decrease over time was reported in Turkish natives and Turkish migrants, with both groups rating fewer ruminations at time AV than time BV (Turkish migrants more so than Turkish natives, yet both *p*s < .001). The age groups <18 or > 17 years old (a proxy for eligibility for vaccination) did not interact with timepoint in any of the subscales. Only a significant three-way interaction of group with timepoint and age group (*b* = −1.18, *p* = .010) in the exaggerated measures item indicated that Turkish immigrants who were younger than 18 reported a significantly higher increase in agreement that the measures against the pandemic were exaggerated. All other groups—irrespective of age—indicated an increase as well, however, not as steep as in older Turkish migrants. This did not apply to ruminations, but in this case age group had an effect in BV but not at AV, with older participants reporting fewer rumination than younger ones. This indicates that the vaccination situation showed very little association with any of the COVID-19 related cognitions we had assessed in the present study.

As in the analyses of mental health outcomes, we ran an additional exploratory analysis again using migrant status (i.e., first, vs. second-generation) as a possible predictor for COVID-19-related cognitions. In Austria, first-generation immigrants reported a higher fear of infecting a family member than second-generation immigrants (*b* = 0.31, *p* = .044). Immigrant status (i.e., migrant vs. native), though, was not associated with any other outcomes regarding COVID-19 related cognitions (all *p*s > .127).

## Discussion

The results of the present study showed that the mental health of young people did not significantly improve in the time period after vaccinations became widely available and promoted in Austria and Turkey. Some previous studies reported that after vaccination the mental health among adults improved (Bilge et al., 2021; Perez-Arce et al., 2021), while these results indicated that at least in Austria and Turkey, there continued to be a decline in the mental health of adolescents younger than 18 years, especially females and those with continued financial difficulties. This was observed through comparing participants below the age of 18 and above the age of 17 years. The COVID-19 pandemic has certainly severely impaired the social life and mental health of younger individuals, as peer group connections, important for adolescent development, were necessarily restricted for many weeks (Hamoda, 2020). In addition, pandemic control measures have led to a substantial loss of the important feeling of autonomy among young people (Fioretti et al., 2020). In the beginning, COVID-19 vaccines were only available for older people and those at risk, but afterwards they were provided for all adults and later also for minors.

The impact of age may be related to more pronounced uncertainty and anxiety among younger groups, as unpredictable pandemic circumstances make it even more difficult for them to plan their future, as they cross the threshold to adulthood and independence. Perhaps they had impatient or high expectations for the immediate post-vaccination period, regarding improvement in safety and social stability, and when confronted with the reality of the ongoing, slowing but not stopping pandemic, they may have become frustrated and disillusioned. Because of these factors, their uncertainty, anxiety, and frustration may continue to persist even with vaccination outreach. Moreover, previous studies indicated that distress caused by the implemented lockdown’s restrictions may have a risk for long-term sequelae in those burdened by lockdowns (Viner et al., 2022).

In line with these results, a number of recent studies have confirmed that younger adults and females in particular suffered from the adverse outcomes associated with the COVID-19 pandemic (Moghanibashi-Mansourieh, 2020; Pieh et al., 2021; Viner et al., 2022). Earlier data indicate that during the COVID-19 pandemic, females more frequently suffered from indicators of mental health problems such as depressive symptoms (Lei et al., 2020; Mazza et al., 2020) and anxiety (Elbay et al., 2020; White & Van Der Boor, 2020) when compared to males (Akkaya-Kalayci et al., 2020; Özlü-Erkilic et al., 2021). In general, most published data also suggests that females might be more vulnerable to developing psychological problems during the pandemic than males (Ahmed et al., 2020; Gao et al., 2020). Similar to our results, the study of Kämpfen et al., 2020 showed that additional financial problems especially led to an overall deterioration in mental health during the COVID-19 pandemic (Kampfen et al., 2020).

The results of the present study showed that all study groups believed that counter-pandemic measures may have been exaggerated, after the main vaccination campaign period. This may be related to the unrealistic expectation that livelihoods would spontaneously revert to normal with the advent of a vaccine, and that widely relaxed lockdown restrictions should have resulted in a faster return to normal life. Because pandemic control measures persisted after the main vaccination period, this likely led to frustration and the perception that such restrictions are exaggerated. It could also be speculated that either the pandemics or the lockdown might have created a basic feeling of vulnerability that was not sufficiently relieved by the development of the vaccines, as they were erroneously viewed as a panacea rather than a first step in the return to “normal” life. This may be especially true for vulnerable groups with a lower health literacy.

Furthermore, concerns relating to the efficacy of the COVID-19 vaccine, in the presence of numerous SARS-CoV-2 variants, are also forming. People are learning that with highly divergent variants, the protective aspect might diminish, similar to previous avian coronaviruses (Darby & Hiscox, 2021). It is also the case that while current vaccines have been shown to provide protection against hospitalization and severe illness, they do not completely safeguard against infection, as evidenced by vaccinated people becoming infected by COVID-19 (Darby & Hiscox, 2021). With prior infectious disease outbreaks, there have been anti-vaccine movements, and the current pandemic is no exception (Hussain et al., 2018).

### Limitations

The present study had some limitations. We collected data via an anonymous online survey, and therefore could not follow up the same exact individuals from T1/T2 (BV) to T3 (AV), as well as combining data from T1 and T2 for a larger pre-vaccination campaign sample. The recruitment at six months post-vaccination availability may also have missed a timepoint where the populace initially experienced a transient improvement in mental health, followed by a return to the pandemic baseline at six months. For both surveys (before and after the vaccination period), we recruited the study subjects in the same way, via the same social media channels and e-mail contacts to institutions working with young people (e.g., youth centers). We therefore assumed that we could recruit similar groups at BV and AV points, but it cannot be excluded that the results of the present study might have been influenced by between-group differences with regard to age, sex, or socioeconomic status. Furthermore, an anonymous online survey can lead to a biased sample due to differences in internet skills or different access to the net in our group compared to other groups. As described in the sample description, the migrants were originating from a wide range of 11 to 45 countries; therefore, it was not feasible to compare them based on their diverse original ethnic backgrounds, but only based on the presence or absence of migration background, without further consideration of other cultural and religious differences. Further, degree of acculturation might be a factor not sufficiently reflected by our basic distinction between first vs. second generation status. Finally, the present study was also limited to data from Austria and Turkey, i.e., one country with a higher and one with a lower income level, therefore the results of the present study might not be generalizable to other countries, as differences in the socio-economic situations or other social factors, but also different lockdown strategies (Akkaya-Kalayci et al., 2020) and vaccination campaigns, or different progress of the pandemics may lead to different outcomes in other countries. As a final point, we could not include sensitive personal information such as vaccination status due to the use of an online survey. However, we still believe that the data did provide important information concerning risk factors and problems in high- and low-income countries during the COVID-19 pandemic and can guide further research and targeted interventions for vulnerable groups and serve to address key problems during the pandemics.

## Conclusions

The impact of the COVID-19 pandemic on mental health and psychological well-being of young people, in Austria and Turkey, was examined in our previous paper (Akkaya-Kalayci et al., 2020), in the initial phase of the pandemic. However, the focus of the present study was to compare the time before and after the COVID-19 vaccination and its impact on mental health and psychological well-being of young people living in Austria and Turkey. To the best of our knowledge, there are so far no published studies conducted that explicitly compare the impact of the COVID-19 pandemic on psychological well-being between migrant and non-migrant Austrian and Turkish young people aged 15–25 years before and after the vaccination campaign. Therefore, the present study, a transcultural comparison of the COVID-19 pandemic between Austria and Turkey, should be considered the first step on this subject.

To overcome the pandemic by preventing the spread of COVID-19 and its adverse outcomes, worldwide vaccination is a critical aspect that will facilitate an eventual return to normal life. It was therefore expected that COVID-19 vaccines would significantly reduce health risks and improve economic and social outcomes associated with the pandemic, and consequently would be beneficial to resolve identified mental health challenges (Perez-Arce et al., 2021). Some studies indicated that COVID-19 vaccination campaigns have had a positive effect on mental health (Bilge et al., 2021; Koltai et al., 2021). However, the results of the present study showed a deterioration in mental health and the psychological well-being of young people, especially in adolescents younger than 18 years, six months after the initial availability of vaccines, noting that in the beginning the vaccine was only available for older groups of adults. Furthermore, the data showed that the beneficial effect of vaccination campaigns might not extend to some vulnerable groups, which were already at a higher risk for pandemic- or lockdown-related mental health problems. Vaccinations alone can improve but not resolve psychological distress and problems, especially in young people and other vulnerable groups. The COVID-19 pandemic had, as confirmed by several of the mentioned studies, a negative impact on the mental health status as well on the socio-economic status of individuals. Many young people and students lost their jobs. As the pandemic continues, these problems will only be prolonged, and the initial erroneous conclusion that vaccines would provide an immediate return to normalcy only serves to increase frustration when faced with the reality of a slow path to recovery. Therefore, low barriers, including online free psychological treatment offers, should be implemented in the healthcare system, in addition to better financial support for economically weak groups by governments to prevent more “collateral” damage caused by both the pandemics and the strict but necessary lockdown measures among the young population. Globally, vaccination campaigns should emphasize the positive effects and benefits of COVID-19 vaccination on general well-being, without creating unrealistic expectations that lead to impatience and discouragement, and must address public anxiety and vaccination hesitancy, especially in the vulnerable groups identified in the present study.

Ultimately, public health messaging must take a two-pronged approach: provide realistic expectations of what the road back to normalcy entails, as well as providing the resources to help the population, especially the vulnerable youth, cope with these trying times. Only then can the government fulfil its duty to protect not only the health of the population, but also the mental health. It is for this reason that we advocate for free access to psychological treatment and financial support.

## Supplementary Information

ESM 1 (DOCX 30 kb)

## Data Availability

The datasets generated during and analyzed during the current study are not publicly available due to privacy but are available from the corresponding author on reasonable request.
