# Supplementary material for: Did the mental health and well-being of young people increase after the COVID-19 vaccination campaign period? A cross-sectional multicentre study in Austria and Turkey
Source: Curr Psychol. 2023 Feb 21:1–14. Online ahead of print. doi: 10.1007/s12144-023-04366-x (PMC9943030; doi:10.1007/s12144-023-04366-x)
Supplement: Supplementary file 1 — (DOCX 30 kb) [file 12144_2023_4366_MOESM1_ESM.docx]

Supplementary Table 1. Descriptive baseline results of the Psychological General Well-Being Index.

|  | **Before Vaccination Campaign Period (*N* = 3665)** | | | | | | **After Vaccination Campaign Period (*N* = 2489)** | | | | | | |  |
| --- | --- | --- | --- | --- | --- | --- | --- | --- | --- | --- | --- | --- | --- | --- |
|  | **Anxiety** | **Depression** | **Personal Well-Being** | **Self-Control** | **General Health** | **Vitality** | | **Anxiety** | **Depression** | **Personal Well-Being** | **Self-Control** | **General Health** | **Vitality** | |
| **Sample Austria (*n* = 2773)** |  |  |  |  |  |  | |  |  |  |  |  |  | |
| Austria Native (*n* = 1902) | 83.50 (22.07) | 91.47 (22.70) | 73.90 (19.69) | 87.03 (23.61) | 96.98 (17.05) | 75.07 (21.08) | | 74.68 (23.38) | 81.51 (25.26) | 66.34 (19.10) | 78.37 (25.57) | 90.02 (20.26) | 64.63 (22.01) | |
| Austria Migrant (*n* = 871) | 82.88 (23.64) | 88.63 (25.76) | 70.16 (21.05) | 85.47 (24.00) | 93.91 (19.96) | 71.67 (23.13) | | 78.02 (23.67) | 83.42 (25.52) | 66.20 (20.30) | 79.84 (24.68) | 88.93 (20.59) | 67.25 (23.27) | |
| **Sample Turkey (*n* = 3381)** |  |  |  |  |  |  | |  |  |  |  |  |  | |
| Turkey Native (*n* = 2704) | 59.55 (21.80) | 67.87 (24.07) | 51.27 (14.98) | 67.73 (25.31) | 75.97 (20.98) | 56.69 (19.39) | | 56.78 (21.68) | 64.90 (23.72) | 49.79 (15.30) | 64.79 (25.07) | 75.01 (20.77) | 54.50 (19.31) | |
| Turkey Migrant (*n* = 677) | 54.75 (19.56) | 64.23 (22.21) | 50.23 (13.43) | 63.78 (23.44) | 71.48 (20.12) | 53.62 (17.36) | | 58.85 (21.18) | 66.57 (24.89) | 50.16 (14.06) | 66.46 (23.20) | 74.69 (18.98) | 56.47 (18.93) | |

Note. Means and SDs. Possible range of each subscale: 20–120.

Supplementary Table 2. Descriptive baseline results of Subscales of the COVID-19 related cognitions.

|  | **Before Vaccination Campaign Period (*N* = 3665)** | | | | | **After Vaccination Campaign Period (*N* = 2489)** | | | | |
| --- | --- | --- | --- | --- | --- | --- | --- | --- | --- | --- |
|  | **Estimated Severity of COVID-19** | **Fear of Being Infected** | **Fear of Infection of a Family Member** | **Belief in Exaggerated Measures** | **Rumination about COVID-19** | **Estimated Severity of COVID-19** | **Fear of Being Infected** | **Fear of Infection of a Family Member** | **Belief in Exaggerated Measures** | **Rumination about COVID-19** |
| **Sample Austria (*n* = 2773)** |  |  |  |  |  |  |  |  |  |  |
| Austria Native (*n* = 1902) | 3.30 (1.07) | 2.09 (1.00) | 3.22 (1.22) | 2.08 (1.16) | 2.82 (1.02) | 3.46 (1.11) | 2.26 (1.13) | 3.26 (1.26) | 2.46 (1.23) | 3.11 (1.05) |
| Austria Migrant (*n* = 871) | 3.20 (1.24) | 2.34 (1.34) | 3.55 (1.34) | 2.32 (1.31) | 2.69 (1.17) | 3.30 (1.16) | 2.30 (1.28) | 3.48 (1.43) | 2.94 (1.34) | 2.75 (1.20) |
| **Sample Turkey (*n* = 3381)** |  |  |  |  |  |  |  |  |  |  |
| Turkey Native (*n* = 2704) | 4.35 (0.83) | 4.02 (1.14) | 4.66 (0.75) | 1.36 (0.82) | 3.59 (1.00) | 4.02 (0.93) | 3.57 (1.22) | 4.32 (0.95) | 2.45 (1.28) | 2.92 (1.06) |
| Turkey Migrant (*n* = 677) | 4.35 (0.86) | 4.05 (1.08) | 4.70 (0.67) | 1.37 (0.87) | 3.74 (0.98) | 4.07 (0.97) | 3.49 (1.23) | 4.35 (1.00) | 2.32 (1.31) | 2.94 (1.04) |

Note. Means and SDs. Possible range for each item: 1–7.
